# United States Veterinary Medical Association

**Published:** 1895-06

**Authors:** 


United States Veterinary Medical Association.—Another annual meeting of our Association will soon be at hand, and no doubt many of our members are awaiting with interest the programme of the meeting, so far as it can be arranged at this early date. That the meeting will be a social success cannot be doubted. That it is to be an intellectual treat past experience leads us to believe. Nothing so improves us as to meet with each other and exchange opinions. Nothing leads to so much good-fellowship and makes of us better individuals. Life would indeed be "not worth the living" were it not for the reward of work in pleasure, and there is no pleasure so great for those who have any desire to learn as that which, under a change of surroundings, and amid old friends and new acquaintances, adds to our store of knowledge in our life's work. It is to be hoped that a large number may be induced to go on to Iowa from the East, so that special cars may carry the members from Boston, to be joined by others from New York, Philadelphia, and Baltimore, and so on to the West. Such an excursion would greatly reduce expenses, besides being of great advantage from a social standpoint. Under no other circumstances is there such an opportunity for mutual exchange of opinions as upon a long railway journey. This Journal would be glad to get the opinion of the members in the East upon this matter and to further in any way such a project.

